# The association of lifestyle and stress with poor glycemic control in patients with diabetes mellitus type 2: a Croatian nationwide primary care cross-sectional study

**DOI:** 10.3325/cmj.2015.56.357

**Published:** 2015-08

**Authors:** Valerija Bralić Lang, Biserka Bergman Marković, Davorka Vrdoljak

**Affiliations:** 1 Private General Practitioner's Office, Zagreb, Croatia; 2 Department of Family Medicine, University of Zagreb, School of Medicine, Zagreb, Croatia; 3 Department of Family Medicine, University of Split, School of Medicine, Split, Croatia

## Abstract

**Aim:**

To assess lifestyle habits and self-reported stress levels among type 2 diabetes mellitus (T2DM) patients and their association with hemoglobin A1c (HbA1c) in general practitioners' (GP) offices in Croatia.

**Methods:**

449 GPs from all Croatian regions from 2008 to 2010 consecutively recruited up to 20-25 participants diagnosed with T2DM at least 3 years prior to the study, aged ≥40 years, and scheduled for diabetes control check-ups. The recruitment period lasted six months. Lifestyle habits and self-reported stress were assessed using the questionnaire from the Croatian Adult Health Survey.

**Results:**

The study included 10 285 patients with T2DM with mean (±standard deviation) age of 65.7 ± 10.05 years (48.1% men). Mean HbA1c level was 7.57 ± 1.58%. 79% of participants reported insufficient physical activity, 24% reported inappropriate dietary patterns, 56% reported current alcohol consumption, 19% were current smokers, and 85% reported at least medium level of stress. Multivariate analysis showed that having received advice to stop drinking alcohol, inadequate physical activity, consumption of milk and dairy products, adding extra salt, and high level of stress were significantly associated with increased HbA1c (*P* < 0.05).

**Conclusion:**

Poor glycemic control was more frequent in patients who had several “unhealthy” lifestyle habits. These results suggest that diabetes patients in Croatia require more specific recommendations on diet, smoking cessation, exercise, and stress control.

According to Croatian National Diabetes Registry, in Croatian population there are 6.3% of registered type 2 diabetes mellitus (T2DM) patients (1). T2DM contributes to the development of circulatory system diseases, which are among ten leading causes of death in Croatia and which cause significant physical disability. As such, it represents a growing burden to health economy. The total treatment cost of T2DM and its complications in Croatia amounted to 11.49% of the Croatian Health Insurance Fund’s budget in 2009, ie, to 351.7 million EUR: 50.2 million EUR for direct medical costs of diabetes treatment and monitoring, 301.5 million EUR for treatment of diabetes-related chronic complications, and 4.6 million EUR for additional indirect costs (2). It is well established that the risk of microvascular and macrovascular complications is related to glycemia. Average blood glucose levels in the previous 3 months are measured by hemoglobin A1c (HbA1c), which still remains a major therapy monitor in diabetes (3). To reduce the incidence of microvascular disease, the American Diabetes Association’s “Standards of Medical Care in Diabetes” recommends lowering HbA1c to <7.0% (4). Since glucose control requires active patients’ participation and commitment, targeted HbA1c treatment must be individualized (5). Given the tremendous disease burden and financial costs associated with diabetes complications, understanding modifiable predictors of diabetes disease course has great public health importance. T2DM patients are often recommended to control body weight, engage in physical activity, and eat a balanced diet. Regular physical activity is beneficial not only for body weight control, but also for improvement of insulin sensitivity and has been reported to reduce the overall mortality of T2DM patients (6). For these patients, it is also important to consume low-fat, low-salt, high-fiber, and low glycemic load diet (7). Another potentially modifiable behavior that may affect the disease course is alcohol consumption. Moderate alcohol consumption has been associated with better glycemic control than abstinence (8). Apart from the association with the development of T2DM, smoking was also related to higher mortality and morbidity (9,10). Substantial data support the theoretical importance of stress in T2DM, yet there is little direct evidence that stress plays a clinically significant role. Higher stress levels showed no prospective relationship with HbA1c, but higher stress levels attributable to diabetes displayed both cross-sectional and longitudinal relationships with HbA1c (11).

According to the Croatian Adult Health Survey (CAHS) 2008, risk factors for T2DM in Croatian adult population were unhealthy nutritional regimen, excessive alcohol consumption, smoking, and lack of physical activity (12). The aim of this study was to investigate the associations between lifestyle habits, self-reported stress, and glycemic control in people diagnosed with T2DM who received medical care in a primary care setting.

## Patients and methods

### Study design, setting and participants

This national, multicenter, cross-sectional study was conducted in Croatian primary care setting between 2008 and 2010. Targeted population were patients of both sexes, diagnosed with T2DM at least three years prior to the study, aged ≥40 years, who visited a primary care practice for diabetes control. The exclusion criterion was the patients’ inability to understand and answer the questionnaire without help. We performed a complex, two-stage sampling. At the first stage, a two-way stratified, random sample of GPs was obtained. Population was stratified according to: 1) the number of insured patients contracted between GPs and the Croatian Health Insurance Fund (national compulsory health insurance system covering 97% of the population) in 2007 (≤1399 patients, 1400-1799 patients, ≥1800 patients) and 2) county (21 Croatian counties). A final sample of physicians was obtained from the list of GPs within each stratum provided by the Croatian National Institute of Public Health and Croatian Health Insurance Fund by using random number generator (13). At the second stage, each GP chose a consecutive sample of patients.

According to power analysis, a sample size of n = 8205 was needed to achieve 95% power with statistical significance level set at <0.05, minimal odds ratio to be considered significant by multivariate, binary logistic regression of OR = 1.1, coefficient of determination of other variables (vital and clinical parameters) for increased HbA1c (≥7.0%) of R2 = 0.3, prevalence of HbA1c ≥7.0% = 50% under the null hypothesis of no association with life-style and stress variables, and two-tail testing of statistical significance. Anticipating approximately 15% of item non-response, the initially needed sample size was determined to be n = 9654. The final sample size was 10 285 patients. The needed sample size was calculated by Power Analysis and Sample Size Software (NCSS, LLC. Kaysville, UT, USA). The recruitment period lasted six months. Patients were informed about the purpose of the study and told that the study participation was anonymous and voluntary. They were asked to fill out the questionnaire on lifestyle habits and stress immediately after the consultation. The questions were the same as those used in the 2003 CAHS (Supplementary material 1 and Supplementary material 2). The most important lifestyle habits were defined as follows: (i) Participants who reported at least two of the following risks were considered as having an unhealthy diet – daily consumption of animal fat, salt-cured meat, milk and dairy products with more than 3.2% of fat, not eating fruit every day or eating it occasionally, always adding extra salt to food. (ii) Participants who reported at least two of the following risk factors were considered as physically inactive – working at home, traveling to work by public transport or working within a 15-min walking or cycling distance, easy or very easy job (sedentary or walking), physical activity of less than 30 min a day during leisure time, having received advice by a health care professional within the past year to increase physical activity. (iii) Participants who reported drinking six or more glasses or bottles of alcohol on a single occasion, once a week or more often, or drinking every day, and who received advice from a family member or health care professional to drink less were considered to be at risk of excessive alcohol drinking. (iv) Participants who smoked at the time of the survey period were considered to be smokers. Those who stopped smoking within five years before the study were considered former smokers. (v) Self-reported stress was measured using Perceived Stress Scale (PSS), which was designed to measure an individual’s appraisal of their life as stressful (14). Item examples include: “How often have you felt nervous or stressed?” and “How often have you felt confident about your ability to handle your personal problems?”. Participants rated how often they had experienced these feelings in the last month on a scale from 0 = never to 4 = very often. PSS-10 scores were obtained by reversing the scores on the four positive items – 4, 5, 7, and 8. Total scores ranged from 0 to 40, with higher scores indicating greater overall stress levels. Three categories of stress were 0-13 indicating low level of stress; 14-26 indicating medium level of stress; 27-40 indicating high level of stress.

After completion of the questionnaire, we made two consecutive measurements of blood pressure, body weight and height, waist circumference, and assessed the presence of comorbidity, taking drugs for diabetes and comorbidities, and HbA1c. HbA1c was measured using an inhibition of latex immunoagglutination with DCA Vantage analyzer from Siemens Healthcare Diagnostics® (Malvern, PA, USA). All measurements were carried out by the GP.

The study protocol was approved by the Medical Ethics Committee of the Medical School, University of Zagreb and the study was conducted in accordance with the Declaration of Helsinki. All participants gave informed consent. To assure anonymity, data were recorded in two different files: one included demographic variables and clinical variables and the other variables related to life style and self-reported stress linked by a consecutive record number.

### Statistical analysis

Univariate, binary logistic regression analysis was used to test the association of physical activity, dietary habits, alcohol consumption, smoking habits, and level of stress (as independent variables) with HbA1c as dependent, binary variable (<7% or ≥7%). Reliability test (Cronbach α coefficient of internal consistency), descriptive statistics, and principal component analysis were used to assess the psychometric properties of the PSS. Significantly associated variables were entered into the multivariate logistic regression model to determine adjusted odds ratios (ORs), while controlling for the possible confounders: body mass index, waist circumference, blood pressure, laboratory measurements, presence of comorbidity, drugs for diabetes, and comorbidities. Statistical significance level was set at α = 0.05 (*P* < 0.05; two-tailed). Statistical analysis was performed using SPSS 17.0 (SPSS Inc., Chicago, IL, USA).

## Results

We studied lifestyle habits and self-reported stress of 10 285 patients with T2DM. Data on HbA1c were available for 10 264 patients. Of 10 335 patients who were asked to participate, 24 patients declined to participate and 26 were excluded for other reasons. There were 4939 (48.1%) men in the sample (Table 1). Male participants were mostly aged between 50 and 69 years (62.6%) and female participants mostly between 60 and 79 years (66.6%). Three quarters of patients reported eating healthy, yet majority of them were overweight – 4226 (40.2%) or obese – 4587 (44.7%). We found no association between overall healthy/unhealthy diet and increased HbA1c except when we analyzed particular dietary habits. A significant association was found between increased HbA1c and consumption of animal fat (*P* = 0.172), milk and dairy products (*P* < 0.001), inadequate fruit consumption (*P* = 0.003), and adding extra salt to food (*P* < 0.001) (Table 2). Majority of patients (8039 or 78.9%) were physically inactive and 6111 or 65.3% were advised by their doctor to increase physical activity. Physical inactive patients were more likely to have increased HbA1c than those who were adequately active (*P* < 0.001). Only 553 (5.5%) patients had ≥150 min/week physical activity, which is recommended for prevention and control of T2DM. Among 3739 (36.4%) working patients, those who spent more time walking or cycling to work were less likely (*P* < 0.001) to have increased HbA1c (Table 3). Wine was most commonly used beverage among participants who reported drinking alcohol (4860/48.9%), followed by beer (3455/35.0%) and spirits (2786/28.0%). 2013 (24.7%) patients were advised by their doctor to stop drinking alcohol. Increased HbA1c was not associated with not excessive alcohol drinking and was significantly associated with having received advice to stop drinking alcohol (*P* < 0.001). More men than women were smokers (23.9% vs 15.2%). Smokers were two times more likely to have increased HbA1c than non-smokers. Majority of patients (7655 or 78.3%) reported medium level of stress, while 1432 (14.7%) and 687 (7.0%) reported low and high levels of stress, respectively. Level of stress was significantly (*P* < 0.001) associated with increased HbA1c (Table 4).

**Table 1:** Characteristics of patients included in the study*

Sex, n (%)		
male	4939 (48.1)	
female	5336 (51.9)	
Age (years), mean±SD	65.7 ± 10.05	
Waist circumference, mean±SD	101.0 ± 13.05	
Elevated blood pressure >140/80 (mmHg), n (%)	8995 (87.5)	
Systolic blood pressure (mmHg), mean±SD	139.0 ± 16.36	
Diastolic blood pressure (mmHg), mean±SD	82.8 ± 8.56	
Total cholesterol (mmol/L), mean±SD	5.45 ± 1.12	
Triglycerides (mmol/L), mean±SD	2.04 ± 1.05	
Creatinine (µmol/L), mean±SD		
male	93.1 ± 21.60	
female	84.8 ± 19.91	
ALT (U/L), mean±SD	28.3 ± 14.81	
Current smoking, n (%)	1969 (19.4)	
Alcohol consumption, n (%)	5861 (57.0)	
Unhealthy diet, n (%)	2398 (23.3)	
Insufficient physical activity, n (%)	8139 (79.1)	
Total level of stress, mean±SD	19.1 ± 5.53	
Level of stress, n (%)		
low	1432 (14.7)	
medium	7655 (78.3)	
high	687 (7.0)	
No of comorbidities, mean±SD	2.0 ± 1.8	
Presence of comorbidity, n (%)	8229 (80.0)	
Drugs for diabetes, n (%)	9913 (96.4)	
Drug for comorbidities, n (%)	8366 (81.3)	
HbA1c, mean±SD	7.6 ± 1.58	
Elevated HbA1c (≥7.0%), n (%)	6031 (58.8)	

*BMI – body mass index; ALT – alanine aminotransferase; HbA1c – hemoglobin A1c; SD – standard deviation.

**Table 2:** Association of type 2 diabetes mellitus patients’ dietary habits with HbA1c ≥7.0%*

	OR_uv_	(95% CI)	*P*
Fats origin			
do not consume fats	1		
herbal	0.94	(0.77-1.27)	0.943
animal	1.17	(0.92-1.57)	0.172
Dairy products/milk			
do not consume milk	1		
skimmed milk	1.17	(0.99-1.38)	0.067
partly skimmed milk	1.25	(1.08-1.46)	0.004
whole milk	1.40	(1.18-1.67)	<0.001
Eating fruit			
every day	1		
very often	1.09	(0.98-1.20)	0.112
occasionally	1.04	(0.94-1.14)	0.487
do not eat fruit	1.40	(1.12-1.75)	0.003
Eating smoked and cured meat products			
do not eat	1		
very rarely	0.97	(0.86-1.08)	0.584
twice a week	1.03	(0.91-1.17)	0.624
every day	1.15	(0.97-1.35)	0.106
Extra salt			
never	1		
sometimes, when not salty enough	0.95	(0.88-1.03)	0.219
always	1.46	(1.2-1.76)	<0.001
Diet			
healthy	1		
unhealthy	0.99	(0.90-1.09)	0.859

*HbA1c – hemoglobin A1c; OR_uv_ – odds ratio, univariate, binary logistic regression; CI – confidence interval.

**Table 3:** Physical activity among type 2 diabetes mellitus patients and its association with HbA1c ≥7.0%*

	OR_uv_	95% CI	*P*
How do you go to work?			
don’t work or work at home	1		
by car or public transport	1.13	1.01-1.25	0.031
walk or cycle	1.29	1.26-1.44	<0.001
Walk or cycle to work			
less than 15 minutes	1		
between 15 and 30 minutes	0.61	0.49-0.78	<0.001
more than 30 minutes	0.47	0.37-0.60	<0.001
Physical activity of at least 30 minutes a day			
inactive	1		
active	1.04	1.01-1.21	0.035
Total physical activity			
adequate	1		
inadequate	1.36	1.23-1.49	<0.001
Received doctor's advice to increase physical activity			
no	1		
yes	0.99	0.91-1.08	0.817
Received other health care worker’s advice to increase physical activity			
no	1		
yes	1.11	1.02-1.22	0.020

*HbA1c – hemoglobin A1c; OR_uv_ – odds ratio, univariate, binary logistic regression; CI – confidence interval.

**Table 4:** Smoking, drinking alcohol, and stress among type 2 diabetes mellitus patients and their association with HbA1c ≥7.0%*

	OR_uv_	95% CI	*P*
Smoking			
no	1		
yes	1.17	1.06-1.29	0.003
Drinking any alcohol			
no	1		
yes	0.95	0.87-1.02	0.179
Drinking spirits			
no	1		
yes	1.07	0.98-1.17	0.158
Drinking wine			
no	1		
yes	0.95	0.87-1.02	0.167
Drinking beer			
no	1		
yes	0.98	0.89-1.06	0.580
Received doctor's advice to stop drinking alcohol			
no	1		
yes	1.50	1.35-1.66	<0.001
Received other health worker’s advice to stop drinking alcohol			
no	1		
yes	1.92	1.70-2.17	<0.001
Received advice from family members to stop drinking alcohol			
no	1		
yes	1.78	1.59-2.00	<0.001
Received anyone’s advice to stop drinking alcohol			
no	1		
yes	1.46	1.32-1.62	<0.001
Level of stress			
low	1		
medium	1.25	1.12-1.41)	<0.001
high	1.33	1.11-1.6	0.003

* HbA1c – hemoglobin A1c; OR_uv_ – odds ratio, univariate, binary logistic regression; CI – confidence interval.

Cronbach’s α reliability coefficient for the stress questionnaire was 0.82, with Guttman’s split-half coefficient 0.85. Principal component analysis with the extraction criterion of eigenvalues >1 revealed two principal components, together explaining 59% of the manifest items variance. Two components were defined by the positive or negative orientation of PSS items.

In univariate analysis, excessive drinking, inadequate physical activity, consumption of milk and dairy products, adding extra salt, and high level of stress were significantly associated with increased HbA1c (*P* < 0.05), which remained unchanged after adjustment for all other variables in the multivariate model (Table 5).

**Table 5:** Lifestyle habits and level of stress and their association with HbA1c ≥7.0%*

	HbA1c	Adjusted model		
	≥7.0% (n = 6031)	OR_mv_	95% CI	*P*
Received anyone’s advice to stop drinking alcohol, n (%)				
no	4508 (56.6)	1		
yes	1523 (66.1)	1.39	1.24-1.57	<0.001
Total physical activity, n (%)				
adequate	1133 (52.8)	1		
inadequate	4898 (60.3)	1.39	1.25-1.56	<0.001
Dairy products/milk consumption, n (%)				
do not consume milk	409 (53.5)	1		
skimmed milk	1314 (57.3)	1.24	1.03-1.50	0.022
partly skimmed milk	3145 (59.0)	1.18	0.99-1.40	0.059
whole milk	908 (61.7)	1.41	1.16-1.72	0.001
Eating fruit, n (%)				
every day	2902 (57.7)	1		
very often	1296 (59.8)	1.10	0.98-1.24	0.101
occasionally	1398 (58.6)	1.02	0.91-1.14	0.775
do not eat fruit	244 (65.6)	1.13	0.88-1.45	0.341
Adding extra salt, n (%)				
never	2836 (58.9)	1		
sometimes	2627 (57.6)	0.95	0.86-1.04	0.252
always	352 (67.6)	1.33	1.07-1.65	0.011
Level of stress, n (%)				
low	771 (54.1)	1		
medium	4554 (59.6)	1.17	1.03-1.33	0.016
high	419 (61.0)	1.29	1.04-1.59	0.018
Current smoking, n (%)				
no	4737 (58.1)	1		
yes	1214 (61.8)	1.06	0.94-1.19	0.349
Confounders controlled				
Sex, n (%)				
male	2864 (58.1)	1		
female	3162 (59.4)	1.25	1.12-1.39	<0.001
Age (years), mean±SD	65.3 ± 10.05	1.00	0.99-1.00	0.235
BMI (kg/m^2^), mean±SD	30.2 ± 4.91	0.99	0.98-1.00	0.098
Waist circumference (cm), mean±SD	101.4 ± 13.06	1.01	1.00-1.01	0.001
Systolic blood pressure (mmHg), mean±SD	139.8 ± 16.74	1.01	1.00-1.01	<0.001
Diastolic blood pressure (mmHg), mean±SD	83.3 ± 8.70	1.00	1.00-1.01	0.202
Total cholesterol (mmol/L), mean±SD	5.5 ± 1.17	1.06	1.01-1.11	0.016
Triglycerides (mmol/L), mean±SD	2.2 ± 1.16	1.34	1.27-1.41	<0.001
Creatinine (µmol/L), mean±SD		1.00	1.00-1.00	0.005
male	93.5 ± 21.83			
female	85.6 ± 20.93			
ALT (U/L), mean±SD	28.9 ± 15.60	1.00	1.00-1.01	0.043
No of comorbidities, mean±SD	1.5 ± 1.26	0.94	0.90-0.97	0.001
Drugs for diabetes, n (%)				
no	201 (54.3)	1		
yes	5830 (58.9)	1.84	1.72-1.98	<0.001
Drug for comorbidities, n (%)				
no	1277 (66.9)	1		
yes	4754 (56.9)	0.69	0.60-0.79	<0.001

*OR_mv_ – odds ratio, multivariate, adjusted, binary logistic regression; CI –confidence interval; ALT – alanine aminotransferase; SD – standard deviation; HbA1c – hemoglobin A1c.

## Discussion

Our study showed that excessive alcohol drinking, adding extra salt, inadequate consumption of fruit, consumption of milk and dairy products, inadequate physical activity, high level of stress, and smoking were significantly associated with poor glycemic control measured as HbA1c >7.0%.

### Alcohol drinking

Previously published data showed less heavy alcohol consumption among diabetics in Croatia compared to our results (12). Receiving anyone’s advice to stop drinking alcohol showed a significant association with HbA1c. Since these patients received advice to stop drinking alcohol both from health care professionals and a family member it is fair to conclude that their alcohol drinking habit was excessive. Presuming so, our results are in agreement with the findings of Ahmed et al (8), who found increased HbA1c among the heaviest drinkers. Otherwise, in their study alcohol drinking habits were not associated with HbA1c. Occasional episodes of alcohol consumption generally do not worsen blood glucose control in people with diabetes and light to moderate alcohol intake (one to two drinks per day; 15-30 g alcohol) are associated with a decreased risk of cardiovascular disease (CVD) (15,16). Therefore, alcohol consumption should not be totally restricted to T2DM patients but also not encouraged in non-drinkers and one should not ignore the dose-dependent risk of alcohol consumption.

### Consumption of salt

There is strong evidence that current consumption of salt is a major risk factor for increased blood pressure (BP) and a modest reduction in salt intake lowers BP regardless of whether BP is normal or raised. Therefore, reduced salt intake is recommended for management of CVD risks in T2DM patients and not only for glycemic control (17). There is no consensus regarding restricting salt intake in diabetic patients. Pooled analysis on the effect of altering salt intake on HbA1c did not show a significant effect of salt intake on HbA1c, although increased HbA1c and dietary sodium intake are suggested to have synergistic effect on development of CVD (18,19). Even though our study was cross-sectional and there was no precise measurement of salt intake, given the large number of participants the finding that T2DM patients who always add extra salt have 30%-50% more chance to have increased HbA1c is very important. It may be suggested that education on salt intake should be incorporated in treatment of diabetic patients.

### Consumption of milk and dairy products

Insulinotropic effect of milk in healthy participants is described in the literature, yet the relationship between dairy intake and glucose metabolism is still not well understood. Inconsistent findings have been reported on the relationship between dairy products consumption and risk of T2DM, as well as the association between milk and dairy intake products and HbA1c levels (20-23). Our findings of increased HbA1c in T2DM patients who consume dairy products indicate the need for further trials to clarify whether a diet free of dairy products or variation in the types of dairy products intake could improve glycemic control. There is no controversy regarding other findings about dietary habits since low fat products and fruit are part of every nutrition recommendation for diabetes. In our study, majority of T2DM patients reported healthy dietary habits. This is in line with previously published findings among diabetic patients in Croatia (12).

### Physical activity

We found more physically inactive participants than did a previous research in Croatia. This is explicable by the fact that participants in our study were older, more often smokers and obese, and with longer disease duration than patients in the previous study (12). Diabetes patients are often refractory to a lifestyle change. Accepting advice about diet and exercise is much more difficult than accepting drug therapy, and not all physicians are fully aware of this problem (24). Both doctors and patients should make much more effort to implement general measures of diabetes management, because structured exercise programs have demonstrated statistically and clinically significant beneficial effect on glycemic control and complication reduction (25).

### Level of stress

Our patients had a mean level of stress of 19.1. This is similar to the findings in diabetic patients (19.35) (26,27) and higher than findings in healthy people in CAHS study (17.46 for men and 18.32 for women) (). Aside from the potential physiological impact, chronic stress may interfere with a person’s capacity to adhere to lifestyle-modifications that are essential to maintaining health. In this study, medium to increased level of stress was significantly associated with the level of HbA1c. Robertson et al found that stress symptoms significantly and independently moderated the relationship between changes in diabetes self-efficacy and post-intervention HbA1c, and suggested that patients with poorly controlled diabetes who had higher levels of stress symptoms may derive greater benefits from self-management interventions known to improve diabetes self-efficacy (28). These results emphasize the importance of regular screening for stress in clinical setting. If patients' stress can be identified and helped, improvements in their overall diabetes control, as well as quality of life, are likely to follow.

### Smoking

The impact of smoking on HbA1c level among T2DM patients is least investigated, and conflicting results were found (29). Our finding that smokers were 1.2 times more likely to have increased HbA1c correspond with the finding from a study with the largest sample size (n = 40648) and adjustment for possible confounders in analysis (30).

### Conclusions

While our study with a large sample of T2DM patients reveals interesting findings on modifiable predictors of diabetes disease course, it has some limitations. First, glycemic control is affected by a complex interplay of factors (eg, adherence to therapy) besides lifestyle and stress. Second, since our study was cross-sectional we were unable to examine the dynamic interplay between changes in lifestyle and stress and HbA1c. Third, lifestyle habits and stress measures were all collected by self-assessment questionnaire, which includes the risk of under- reporting or recall bias. However, this was taken into account in our data analysis.

In conclusion, our study suggests that doctor's counseling on healthy lifestyle in diabetics should include more specific recommendations on diet (concerning alcohol, salt, milk, and dairy products consumption), smoking cessation, exercise, and stress control.

## 

Accepted: July 15, 2015
